# Isolated cerebellar stroke in a paediatric patient with typical haemolytic uraemic syndrome: a case report and literature review

**DOI:** 10.1007/s00234-024-03407-x

**Published:** 2024-06-26

**Authors:** Manuela Lo Bianco, Sergio Rinella, Felice D’Arco, Evangelia Ioannidou, Marios Kaliakatsos

**Affiliations:** 1 Postgraduate Training Program in Pediatrics, Department of Clinical and Experimental Medicine, University of Catania, Catania, Italy; 2 Department of Developmental Neurosciences, UCL Great Ormond Street Institute of Child Health, University College London, London, UK; 3 Department of Educational Sciences, University of Catania, Catania, Italy; 4 Department of Developmental Neurosciences, UCL Great Ormond Street Institute of Child Health, University College London, London, UK; 5 Department of Neuroradiology, Great Ormond Street Hospital for Children, London, UK; 6 Paediatric Specialty Trainee in Paediatric Neurology, Great Ormond Street Hospital for Children, London, UK; 7 Department of Neurology, Great Ormond Street Hospital for Children, London, UK

**Keywords:** _1_Cerebellum, _2_Posterior-Inferior Cerebellar Artery, _3_PICA, _4_HUS, _5_Case report, _6_Ischaemic injury

## Abstract

Haemolytic Uraemic Syndrome (HUS) is a rare medical condition characterised by microangiopathic haemolytic anaemia, thrombocytopenia, and acute kidney injury. Neurological complications are documented but rarely involve the cerebellum. We present a unique case of a 23-month-old male with HUS triggered by *Escherichia coli*-O157 (*E.coli-*O157) infection leading to an isolated cerebellar stroke.

The patient initially presented with fever, bloody stools, and seizures. Confirmation of *E.coli-*O157 infection was obtained, and MRI revealed an isolated cerebellar stroke. Treatment included supportive care, anticoagulation for a right atrial thrombus, with gradual improvement observed.

This case highlights the unusual occurrence of isolated cerebellar stroke in HUS patients, emphasising the importance of promptly recognizing manifestations of the central nervous system and the necessity for a multidisciplinary approach. Finally, a comprehensive literature review was conducted to identify cases of HUS patients with cerebellar involvement.

## Introduction

Haemolytic Uraemic Syndrome (HUS) is a rare medical condition characterised by microangiopathic haemolytic anaemia, thrombocytopenia, and acute kidney injury. Neurological complications occur in approximately 10–50% of cases and have been well documented [1]. However, cerebellar stroke involvement is an exceptionally uncommon presentation with very limited reported cases. We present a unique case of a 23-month-old male who developed an isolated cerebellar stroke as a complication of HUS triggered by *Escherichia Coli (E.Coli)* O157 infection. This case highlights the rarity and complexity of such cases, emphasising the importance of early recognition and multidisciplinary care.

## Case report

A previously healthy full-term 23-month-old male, born by vaginal delivery, presented to the local hospital with a 5-day history of pyrexia and bloody loose stools. The child was up to date with immunisation and had no history of travel. Initial diagnostic concerns included intussusception, therefore he was transferred to a tertiary paediatric surgical unit, where this diagnosis was excluded, and the patient discharged. A stool sample confirmed the presence of *E.Coli-*O157, and an ongoing *E.Coli-*O157 outbreak was reported at his nursery. Twenty-four hours after discharge, he re-presented to the local Emergency Department following a generalised tonic–clonic (GTC) seizure. He was also noted to have oligo-anuria. He also experienced clusters of GTC seizures, which persisted despite the administration of two doses of benzodiazepines. His blood gas revealed metabolic acidosis with marked hyponatraemia and his blood test confirmed mild anaemia, deranged kidney function, elevated liver enzymes, and a markedly elevated C-reactive protein. Continuous veno-venous hemofiltration, hypertonic (3%) saline and diuretics were administered in view of acute kidney injury with metabolic acidosis.

The patient was urgently intubated for neuroprotection and a brain computed tomography scan was performed to exclude intracranial bleeding which was negative.

Subsequently, he was transferred to the Paediatric Intensive Care Unit at Great Ormond Street Hospital for Children, London, UK, with suspected of HUS. At the time of arrival the patient was neurologically unassessable as was intubated and paralysed.

**Fig. 1:**
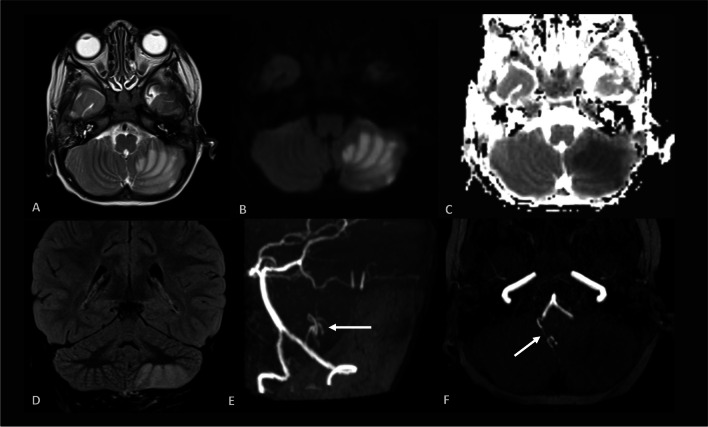
MRI brain scan showing left PICA acute ischemic stroke. The ischaemic area is hyperintense on axial T2 weighted images (A), shows diffusion restriction on axial diffusion weighted images (B) and apparent coefficient diffusion maps (C) and is hyperintense in coronal FLAIR (D). Magnetic resonance angiography, 3D maximum intensity projection (MIP) and axial MIP (E and F) show presence of the right PICA only (arrow) without visualisation of the left one

He underwent a cardiac echo which showed right internal jugular and right atrial (RA) thrombus so he was placed on a heparin infusion. There was no evidence of right to left communication in the heart. His magnetic resonance imaging (MRI) and angiography brain scan revealed an acute left posterior-inferior cerebellar artery (PICA) stroke, showing diffusion restriction, and a reduction in the flow signal of left PICA (Fig. 1). His electroencephalogram was suggestive of diffuse encephalopathic process.

A diagnosis of isolated cerebellar stroke secondary to HUS was made. The patient was treated conservatively and showed gradual clinical improvement, with resolution of seizures, improvement of renal function and urine output. A repeat cardiac echo was unremarkable with no evident thrombus and the child was discharged home three weeks after his initial presentation, on low molecular weight heparin and amlodipine, with close monitoring of factor Xa. At the time of discharge and at review one month later the patient was neurologically normal.

## Discussion

This case report delves into the rare occurrence of isolated cerebellar stroke in a paediatric patient with typical HUS triggered by *E.Coli*-O157 infection.

Shiga toxin, produced by disease-causing strains of bacteria (e.g. *E.Coli-*O157), plays a pivotal role in organ damage. Once released, the toxin is absorbed into the bloodstream through the gastrointestinal tract and binds to globotriaosylceramide (Gb3) on the surface of vascular endothelial cells [2]. This binding triggers a cascade of pathophysiological events by promoting inflammation, inducing the expression of cytokines and chemokines, and eliciting a ribotoxic stress response. Consequently, endothelial cell damage leads to the formation of microthrombi, activation of the alternative complement pathway, and injury to target organs, especially kidney and brain.

Damage to the CNS primarily contributes to mortality in individuals with acute HUS. This brain damage is probably due to the occurrence of microvascular damage in susceptible brain regions, such as brainstem and basal ganglia. Notably, Gb3 has been detected in various types of neurons in murine models, including the cerebellum [3]. Cerebellum is characterised by a unique neurovascular architecture whose main feeders are the superior cerebellar artery, the anterior inferior cerebellar artery, and the posterior inferior cerebellar artery (PICA). The cerebellar vasculature, while intricate, is less susceptible to thrombosis compared to the densely vascularized basal ganglia. However, when a cerebellar stroke occurs, it predominantly manifests at the level of the PICA, with a 40% prevalence [4].

Hence, the most reasonable explanation for the occurrence of cerebellar stroke in the present case could be the underlying vascular changes due to endothelial injury/inflammation in the left PICA territory. However, the occurrence of isolated cerebellar involvement remains extremely rare and the pathogenesis unclear.

Lee et al. [5] reported that isolated cerebellar stroke could originate when cardioembolism occurs in the context of a specific angulation of the PICA. In fact, in the majority of cases, strokes involving the PICA are attributed to cardioembolism [4]. Our patient developed a RA thrombus as a consequence of HUS, which could represent a possible factor bringing to the isolated cerebellar stroke via distal emboli. Nevertheless, there were no other emboli present and two cardiac echos showed no evidence of intra-atrial communication.

A comprehensive literature review was conducted to identify cases of HUS patients with cerebellar involvement. Our search revealed only six cases in which the cerebellum along with other brain areas was implicated in haemorrhagic/ischaemic events associated with HUS (Table 1) [6–10].

**Table 1:** Overview of the cases retrieved from the scientific literature

Reference	Type of study	No. of patients^a^	Sex/Age (years)	Clinical/Laboratory Findings	MRI	Other instrumental examination	Treatment/management	Follow-up MRI	Clinical course
*Gawlitza *et al*., 2015*	Case report	1	F/28	Weight loss, multiple hematomas, anuria, disorientation, progressive confusion Thrombocytopenia, haemolysis, anuric renal failure	FLAIR-hyperintense lesions with restricted diffusion in the centre of the SCC, in both cerebellar hemispheres (dentate nucleus), and in the middle cerebellar peduncle	/	Eculizumab	No signs of restricted diffusion but a slight cerebellar volume loss	Rapid improvement of neurological, haematological and nephrological symptoms
*Steinborn *et al*., 2004*	Case series	1	*/* *(paediatric age)*	Diarrhoea, seizures, coma, hemiparesis	Severe changes, haemorrhage, contrast enhancement in the basal ganglia and in the cerebellum	EEG: distinct generalized changes	/	Reduction in basal ganglia and cerebellum abnormalities (severe to distinct changes)	Weakness in minute motor activity
*Mewasingh *et al*., 2003*	Case series	1	F/5	GTC seizures, bloody diarrhoea, pyrexial (39 °C), hypovolemic shock, generalized hypotonia, dysarthria, dysmetria, action tremor, ataxic gate; stopped vocalizing, unable to sequence symbolic activities, slowness of simple tasks Thrombocytopenia, DIC, acute renal failure	**FLAIR diffuse hyperintensity of the cerebellar cortex**	Abdominal US/colonoscopy: pancolitis; EEG: normal	Haemodialysis; surgical resection a week after recurrence of her diarrhoea	Postoperative MRI: moderate cortical and subcortical atrophy with no abnormal signal in the cerebellar cortex	Deceased 48 h later after a massive pulmonary haemorrhage. Post-mortem sectioning of the brain: massive fresh haemorrhages in ventricles, telencephalic, diencephalic, brainstem, and cerebellum regions. Previous ischemic cerebellar signs, namely, ischemic cerebellar cortical sclerosis with total loss of Purkinje cells and marked loss of neurons in the granular layer
*Nakamura *et al*., 2003*	Case report	1	F/22	Fever, abdominal pain, bloody diarrhoea, GTC seizures, encephalopathy, coma Thrombocytopenia, haemolytic anaemia, oliguric renal failure; stool culture: VTEC, O-157; CSF: slight increase in cell counts and high level of MBP. Brain biopsy: oedematous changes and mild astrocytosis without definite thrombosis and inflammation	Symmetrical T1-iso/T2-hyperintensity signal lesions in midbrain tegmentum and cerebellar vermis	/	Haemodialysis, plasmapheresis, transfusion of RBC	Bilateral and symmetrical increased T2 signal in basal ganglia, occipital lobe, and cerebellar hemisphere. Remarkable reduction of the lesions 70 days after admission	Improved level of consciousness, tetraparesis, limb ataxia, bilateral partial visual field defect
*Hager *et al*., 1999*	Case series	2	M/2.5; M/4	1. Fever (39.6 °C); slightly dehydrated, pale, hypotonic, convulsions, alteration of consciousness, cyanosis, nystagmus, coma Thrombocytopenia; stool culture: VTEC, O-157 2. Fever (38.8 °C); non-bloody diarrhoea, vomiting, severely dehydrated, hypotension, anuria, febrile convulsion, intermittent tonic seizures; right-sided hemiparesis, ptosis, miosis, retinal microangiopathy Thrombocytopenia; stool culture: VTEC, O-157	1.Bilateral signal intensity changes in dorsal putamen, pallidum, right lower cerebellar hemisphere 2. Bilateral signal intensity changes in the dentate nuclei and adjacent white matter, and in the right cerebellar cortex	1. CT-scan: hypodensity of the basal ganglia 2. CT-scan: small lesions in the left basal ganglia. EEG: diffuse delta slowing	1. RBC and PLT transfusion, albumin; BDZ, phenobarbital, PLEX, mechanical ventilation 2. RBC and PLT transfusion; diazepam/phenobarbital-resistant; thiopental; intubation, haemodialysis/peritoneal dialysis, PLEX, albumin	1. Normal MRI at 5-months follow-up 2. Bilateral pseudo-cystic lesions confined to the putamen and T2 signal increased in the cerebellum hemisphere at 3-months follow-up MRI	1. Discharged without neurological symptoms 2. No neurological symptoms after 12 months

*BDZ* Benzodiazepines, *DIC* disseminated intravascular coagulation, *FLAIR* Fluid Attenuated Inversion Recovery, *GTC* Generalised Tonic–Clonic Seizures, *MBP* Myelin Basic Protein, *MRI*, Magnetic Resonance Imaging, *PLEX* Plasma Exchange, *PLT* Platelets; RBC Red Blood Cells, SCC Splenium of the Corpus Callosum, *US* Ultrasound, *VTEC* VerocytoToxin-producing Escherichia Coli

^**a**^Only patients with cerebellar involvement were included

Only one case involved a 5-year-old patient with typical HUS who initially exhibited an isolated cerebellar ischemic stroke [8]. However, HUS was not associated with *E.Coli-*O157 and the report lacked any MRI images for review or mention of the territorial involvement. Unlike our isolated cerebellar presentation, she experienced subsequent haemorrhages in additional cerebral areas.

In conclusion, we report a rare case of an isolated cerebellar stroke in a child with typical HUS triggered by *E.Coli-*O157 infection, suggesting the underlying endothelial injury and/or inflammation in cerebellar vessels can be part of the spectrum of CNS manifestations in patients with HUS.

Finally, effective management of acute HUS necessitates a multidisciplinary approach involving paediatricians, nephrologists, neurologists, haematologists, and psychologists, as early recognition and treatment is crucial for a good outcome.

## Data Availability

The data used in this study are available from the corresponding author upon request.
